# Supplementary material for: The Systems Biology Research Tool: evolvable open-source software
Source: BMC Syst Biol. 2008 Jun 29;2:55. doi: 10.1186/1752-0509-2-55 (PMC2446383; doi:10.1186/1752-0509-2-55)
Supplement: Additional file 1 — SBRT Archive. An archive of the current version of the Systems Biology Research Tool. [file 1752-0509-2-55-S1.zip › sbrt-1.4.0/doc/developers_guide/api/sbrt/kernel/math/algebra/class-use/MatrixElement.html]

Uses of Class sbrt.kernel.math.algebra.MatrixElement


|  |  |  |
| --- | --- | --- |
| **Overview** \| **Package** \| **Class** \| **Use** \| **Tree** \| **Deprecated** \| **Index** \| **Help** | |  |
| PREV   NEXT | **FRAMES**    **NO FRAMES**     **All Classes** |  |


---


## **Uses of Class sbrt.kernel.math.algebra.MatrixElement**

| Packages that use MatrixElement | |
| --- | --- |
| **sbrt.shell.text.math** | Provides classes and interfaces for defining formats for mathematical objects. |

Uses of MatrixElement in sbrt.shell.text.math

Methods in sbrt.shell.text.math with type parameters of type MatrixElement

`<S extends MatrixElement<R,C,java.lang.Double>>
  
java.lang.String`

`MatrixElementFormatV1.format(S indexPair)`
  
          Returns a formatted string representation of the
provided matrix element.

| Methods in sbrt.shell.text.math that return MatrixElement | |
| --- | --- |
| `MatrixElement<R,C,java.lang.Double>` | `MatrixElementFormatV1.parse(java.lang.String element)`             Parses the provided string and returns its corresponding matrix element. |

| Methods in sbrt.shell.text.math with parameters of type MatrixElement | |
| --- | --- |
| `java.lang.String` | `MatrixElementFormatV1.formatElement(MatrixElement<? extends R,? extends C,java.lang.Double> element)`             Returns a formatted string representation of the provided matrix element. |

---


|  |  |  |
| --- | --- | --- |
| **Overview** \| **Package** \| **Class** \| **Use** \| **Tree** \| **Deprecated** \| **Index** \| **Help** | |  |
| PREV   NEXT | **FRAMES**    **NO FRAMES**     **All Classes** |  |


---
